# Pulmonary Artery Hypertension and Related Complications Associated to Left Atrial Myxoma

**DOI:** 10.2174/1874192401711010156

**Published:** 2017-12-21

**Authors:** Osmar Antonio Centurión, Laura Beatriz García

**Affiliations:** Asuncion National University, Division of Cardiovascular Medicine. Clínic Hospital. Av. Mariscal López e/ Coronel Cazal. San Lorenzo, Paraguay; Asuncion National University, Division of Cardiovascular Medicine. Clínic Hospital. Asuncion, Paraguay

Atrial Myxoma (AM) is a primary benign tumour of the heart that may behave in a not so benign manner causing serious cardiac and systemic complications [1-4]. The global annual incidence of AM is 0.5 per million people and may occur in different locations within the heart cavities but primarily affect the left atrium in 75% of the cases [5-7]. There have been reports of myxoma in the right atrium and in the left or right ventricles [8, 9]. The symptoms may widely differ depending on several factor related to the mass itself. AM tends to differ in shape, size, and texture, and originate from the vicinity of the fossa ovalis in the inter-atrial septum. AM occurs in patients between the ages of 30 and 60 years, but it can be seen at any age [7]. Sometimes, AM is discovered incidentally while investigating non-specific symptoms on a routine echocardiography. Most AM are isolated, and they present in familial cases in less than 10%.

The clinical presentation varies among patients and certainly depends on the size, mobility and location of the AM. It can be asymptomatic, or present non-specific symptoms which make early diagnosis difficult. Meng et al. reported a 4% rate of asymptomatic left AM in a series of 149 cases [4]. However, the typical symptoms involve systemic embolism, heart failure, and pulmonary artery hypertension. Systemic embolism occurs in 25% to 50% of left AM and about half of them travel to the central nervous system [5-7]. The clinical diagnosis of AM is based on imaging methodology like echocardiography, cardiac computerized tomography or cardiac magnetic resonance imaging. These imaging techniques provide detailed information about de structure of the tumor mass, its mobility, size, and myocardial invasion. The histopathological study of the cardiac mass confirms the diagnosis of AM [10-13]. These tumors are composed of scattered cells within a mucopolysaccharide stroma. Usually, typical atrial myxomas are pedunculated and gelatinous in consistency; the surface may be smooth, villous, or friable. Tumors vary widely in size, ranging from 1 to 15 cm in diameter, and weighing between 15 and 180 g. About 35 percent of myxomas are friable or villous, and these have a tendency for systemic embolism. Larger tumors are more likely to have a smooth surface and to be associated with heart failure and pulmonary artery hypertension [7].

In patients with left AM, symptoms of left-sided heart failure, such as dyspnea on exertion, may progress to orthopnea, paroxysmal nocturnal dyspnea or pulmonary edema because of obstruction at the mitral valve orifice and pulmonary hypertension [14, 15]. Sometimes, despite the optimization of the medical treatment utilized to ameliorate the signs and symptoms of the heart failure and the pulmonary artery hypertension of these patients there is no improvement in the clinical condition. Therefore, medical therapy may not be enough to ameliorate the symptoms. Evidently the large and mobile left AM generate a mitral valve stenosis-like mechanical obstruction with concomitant left atrial dilatation and pulmonary hypertension which usually disappear after surgical resection of the large atrial mass.

The surgical treatment consists of the total resection of the intra-atrial mass which should be prompt enough and complete to avoid embolism and other cardiac complications [4-6]. When an AM is diagnosed with any imaging methodology it usually implies immediate consequent surgical excision to prevent embolic events. Therefore, studies with documented growth rate are very rare to find, and the actual growth rate remains a controversial issue. Large myxomas may remain asymptomatic if the tumor growth is very slow. AM produces vascular endothelial growth factor, which probably contributes to the induction of angiogenesis and the early stages of tumor growth [16-20]. Walpot J, et al. reported the growth of a left AM in an asymptomatic 65-year-old patient who had several years of close follow-up for the evaluation of aortic valve disease. The calculated growth rate was estimated to be an average growth rate of 0.49 cm/month [21]. Hence, the growth rate of atrial myxomas may be faster than is usually believed. The knowledge of this relatively fast growth of AM has clearly therapeutic implications. Surgery should be done as prompt as the diagnosis is made. Survival after total resection of the AM is high. Nevertheless, since there is a 1% to 3% recurrence long-term follow-up with echocardiography is highly recommended [1-3]. The causes of recurrent AM are inadequate resection, multifocal pattern behavior of a benign tumor with multiple growing in different locations, and seeding of tumor fragments during surgery. Therefore, a total resection of the tumor stalk and its attachment is recommended, especially when the tumor originates from the fossa ovalis. Reber D, et al. reported a recurrence case that was believed to be due to totipotent multi-centricity of the tumor [22]. Familial disposition may also play a role in recurrent development. The abnormal DNA ploidy pattern of myxoma patients showed a high recurrence [22].

Although concurrent primary cardiac tumors are rare, the possibility of additional tumors requires thorough detailed preoperative evaluation in any patient who has a primary cardiac tumor. Preoperative evaluation with cardiac magnetic resonance imaging would help the cardiothoracic surgeon to identify additional tumors and to plan the surgical approach accordingly. Although a left AM can be approached less invasively via a right thoracotomy, the presence of concurrent tumors would require a medium sternotomy for adequate and complete surgical excision. It is not difficult to make the differential diagnosis with current auxiliary diagnostic methods [23-26]. It is now imperative to do genetic studies and also perform imaging studies to the relatives of patients having AM to rule out additional occult familial cases. Once the diagnosis of AM is performed, a surgical resection as early as possible should be done in order to avoid heart failure, pulmonary artery hypertension, and other systemic complications in asymptomatic patients.
